# The relationship between openness and social anxiety: the chain mediating roles of social networking site use and self-evaluation

**DOI:** 10.1186/s40359-023-01412-y

**Published:** 2023-11-13

**Authors:** Jian Gong, Ye Li, Bingyu Niu, Xiaofei Liu, Yuyan Wang, Bingping Zhou, Man Hai

**Affiliations:** 1 Key Laboratory of Adolescent Cyberpsychology and Behavior (CCNU), Ministry of Education; School of Psychology, Central China Normal University, No. 152 Luoyu Street, Hongshan District, Wuhan, Hubei 430079 China; 2 School of Psychology, Inner Mongolia Normal University, No. 81 Zhaowuda Road, Saihan District, Hohhot, 010022 Inner Mongolia China

**Keywords:** Openness, Social anxiety, Active social networking site use, Passive social networking site use, Self-evaluation

## Abstract

**Background:**

As social networking sites (SNSs) with diverse functions gradually become an important social place for modern people, openness, as a personality trait that represents the willingness to consider diverse things, will be more likely to affect people’s cognitive and emotional experience (e.g., social anxiety) in social interactions. This study examined the relationship between openness and social anxiety and the underlying psychological mechanism in the internet age based on the cognitive-behavioral model of social anxiety.

**Methods:**

This cross‑sectional survey study conducted a questionnaire survey of 522 college students from two provinces in China (191 male; age range 18–25; *M* = 20.76, *SD* = 1.34).

**Results:**

The results showed that openness is negatively related to social anxiety. Self-evaluation and passive SNS use independently mediate the relationship between openness and social anxiety, respectively. Moreover, openness is associated with social anxiety both through the chain mediating roles of active SNS use and self-evaluation and through the chain mediating roles of passive SNS use and self-evaluation.

**Conclusions:**

Openness is negatively associated with social anxiety, and the different ways of SNS use and self-evaluation are the underlying mechanisms. These results provide insights into the clinical treatment of social anxiety and how to benefit from online interactions.

**Supplementary Information:**

The online version contains supplementary material available at 10.1186/s40359-023-01412-y.

## Introduction

Social anxiety, one of the most common anxiety disorders, has yet to be treated effectively [1]. Personality trait approaches have great potential in psychotherapy, especially for social disorders [2]. Neuroticism and extraversion personality significantly predict social anxiety in offline interactions [3]. However, with the development of Internet technology, the form of social activities is changing, which also brings new challenges to treating social anxiety [4]. Openness, which has been neglected by previous researchers, plays an increasingly important role in the development of social anxiety [3, 5].

**Fig. 1:**
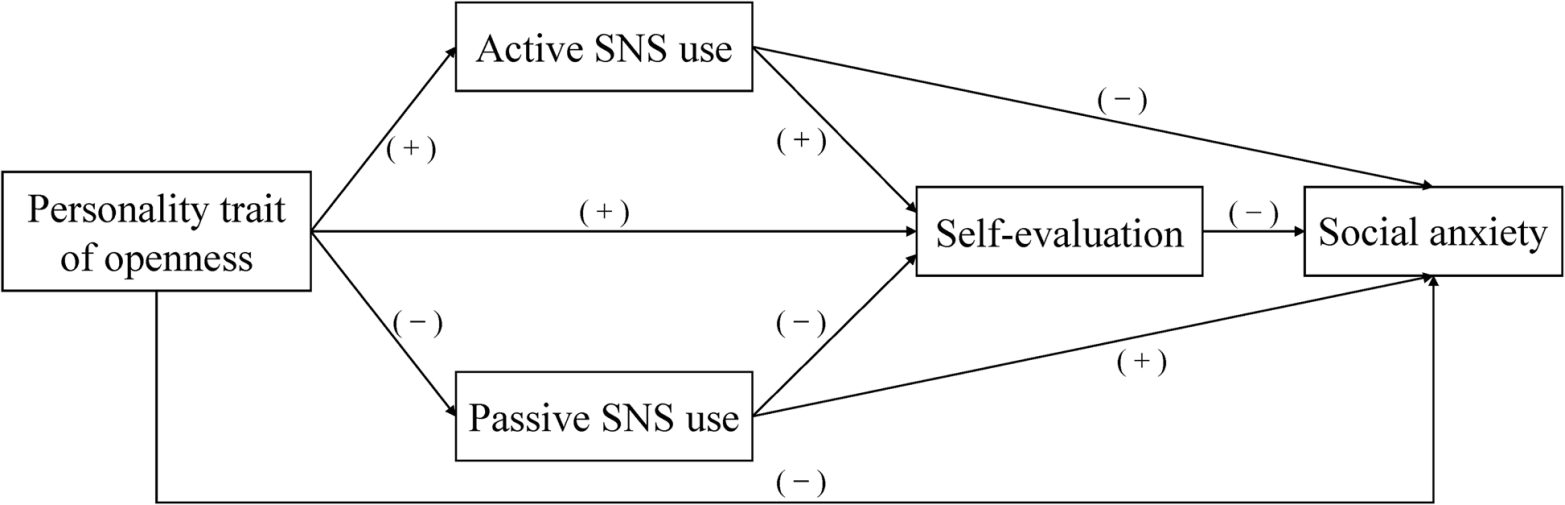
The hypothetical model

Therefore, the current study aimed to investigate the relationship between openness and social anxiety, and whether different ways of social networking site (SNS) use and self-evaluation are the underlying mechanisms (see Fig. 1 for the hypothetical model). The study may help to understand the origin of social anxiety from the cognitive perspective of personality traits, and also deepen the understanding that different ways of SNS use play different roles in the formation of psychopathological consequences.

## Literature review and hypothesis

### Openness and social anxiety

Social anxiety refers to the nonadaptive emotional experience of an individual caused by imagined or real social situations in the process of social interaction, such as feeling uncomfortable, unnatural, nervous, fearful, etc. [6]. According to the cumulative risk hypothesis, certain personality traits place individuals at long-term risk of being more prone to psychological disorders [7]. Conversely, some personality traits can protect individuals from psychological disorders. Openness, as a personality trait that describes a person’s desire for adventure, curiosity for new things, and need for diversity, may be a protective factor for social anxiety.

It is well-known that high openness individuals enjoy seeking different and exciting experiences or sensations [8]. These behavioral styles help protect high openness individuals from social anxiety [9, 10]. In contrast, low openness is related to right-wing authoritarianism [11], religious fundamentalism [12], and other conservative values [13], which are related to prejudice or discrimination [14]. In other words, low openness individuals are more inclined to assert themselves and have difficulty tolerating values that differ from them. Therefore, they are more likely to perceive some social contextual cues as threatening or exaggerate the threat’s extent, which further leads to anxiety [14, 15]. In addition, from a physiological perspective, high openness individuals have better physiological flexibility and the ability to maintain homeostasis, so the physiological stress reactivity in facing social stress is relatively low [16]. Physiological studies of social anxiety also show that individuals with low social anxiety have lower physiological stress reactivity in facing social stress and faster physiological response recovery after the stress [17]. Therefore, the present study proposes the following hypothesis:

- **Hypothesis 1.** Openness is negatively correlated with social anxiety.

### The mediating role of SNS use

Since social networking sites (SNSs) are richer in functions and content than other sites, people increasingly prefer to use SNSs to present themselves, access information, and make friends [18]. Previous studies have shown that high openness individuals are more likely to use SNSs [19–22]. However, it has also been found that openness has little or no correlation with SNS use [23, 24]. This may be due to the fact that previous studies have confused the two ways of SNS use: active and passive. Active use refers to information-generating behaviors that enhance communication, such as posting status updates or comments [25]. Passive use refers to information-browsing behaviors that lack communication, such as viewing others’ homepages or photos [25].

High openness individuals are likely to have higher active SNS use and lower passive SNS use. High openness individuals are more curious about other people’s opinions and unfamiliar situations [8, 26], so they may be more willing to actively interact with others on SNSs rather than being passive viewers. Empirical studies have also shown that high openness individuals actively post personal information, leave comments on friends’ posts, and give thumbs up more often on SNSs [27, 28]. High openness individuals use SNSs to learn about others’ lives and plans, mainly to supplement real-life interactions [29]. In other words, high openness individuals may like to present themselves and interact with others on SNSs, but do not like to monitor others’ lives through SNSs passively.

Active SNS use may be associated with lower social anxiety. Active SNS use can help individuals receive more social support, improve friendship quality, and thus reduce stress and anxiety [30–33]. Furthermore, the characteristics of online interactions can also reduce the likelihood of social anxiety occurring. On the one hand, regarding the virtual nature of the Internet, online interactions can reduce some embarrassing social situations and provide a sense of control that is not often felt in face-to-face interaction [34]. On the other hand, regarding the asynchronous nature of the Internet, SNSs create good conditions for individuals to modify their self-expression, which helps to reduce potential anxiety that may be caused by negative expectations [35].

Conversely, passive SNS use may be associated with higher social anxiety. People tend to portray themselves in overly flattering ways on SNSs [25], which may lead passive SNS users to unconsciously fall into upward social comparisons dilemma when they see their friends’ updates. According to the theory of social comparison, the contrast effect produced by upward social comparison reduces individuals’ self-evaluation and impacts their mental health [36]. Previous studies have also found that individuals who passively use SNSs have higher jealousy and lower self-esteem [36, 37]. In particular, passive SNS use can lead to ruminative thinking related to interpersonal relationships, which easily exacerbates social anxiety symptoms [38, 39].

Overall, the present study hypothesized that openness and social anxiety partially linked via active and passive SNS use. Specifically, we propose:

- **Hypothesis 2.** Active SNS use would mediate the link between openness and social anxiety.
- **Hypothesis 3.** Passive SNS use would mediate the link between openness and social anxiety.

### The mediating role of self-evaluation

Self-evaluation is an individual judgment of the relevant abilities and social adaptability that the self has and is the social cognitive of the self [40]. In general, individuals with low openness have lower self-evaluations. Because they always reject challenges and diverse tasks, which leads them to lose the opportunity to improve self-evaluation through successfully completing such tasks [41]. Previous study has also shown that low openness is significantly correlated with low core self-evaluation [42]. Unfortunately, low self-evaluation may induce social anxiety. According to the cognitive-behavioral model of social anxiety, low self-evaluation is the core cause of social anxiety symptoms [43]. Moscovitch [44] also noted that in the exposure therapy for social anxiety disorder, the stimulus that truly frightens patients is their own self-evaluation rather than the negative feedback from others. Therefore, we propose:

- **Hypothesis 4.** Self-evaluation would mediate the link between openness and social anxiety.

### The chain mediating roles of SNS use and self-evaluation

Nowadays, SNS platforms have been well integrated into modern human life and have become an important way for people to make social comparisons and self-evaluations. Research has suggested that young people are more inclined to obtain other people’s information through SNSs and use this information as the main basis for self-evaluation [45]. In other words, SNS usage has the function of shaping self-evaluation [46] and developing self-concept [47, 48].

When actively using SNSs, individuals can comfortably portray themselves, manage impressions, and receive positive feedback [49]. In addition, individuals are more confident in their ability to maintain a large number of weak ties [32]. As a result, individuals can more effectively accumulate social capital, meet relatedness needs, and develop a positive self-perception.

Conversely, individuals who passively use SNSs lack self-disclosure and communicative interaction, which hinders the establishment and development of high-quality relationships with others [25]. Therefore, passive SNS users are more likely to be maladjusted [25]. In addition, individuals who passively use SNSs passively accept the “perfect” image of others for a long time, but cannot obtain good evaluations of themselves from others. Such perceptions of positive images about others and negative images about themselves may be detrimental to self-evaluation. Therefore, we propose:

- **Hypothesis 5.** Active SNS use and self-evaluation play chain mediating roles in openness and social anxiety.
- **Hypothesis 6.** Passive SNS use and self-evaluation play chain mediating roles in openness and social anxiety.

## Materials and methods

### Participants

Participants included 571 students from Shanxi Province and Hubei Province in China who were selected using convenience sampling. Excluding the participants whose response time was less than 180 s (31 participants) or failed attention-check items (18 participants), 522 valid questionnaires were finally recovered (the effective recovery rate was 91.42%). All participants were college students (191 male; age range 18–25; *M*_age_ = 20.76, *SD*_age_ = 1.34).

### Procedure

This is a quantitative cross-sectional study. The data collection was conducted from May to July 2022. The study recruited 571 participants from two universities in Shanxi Province and Hubei Province (in northern and southern China, respectively). An online survey was created using Credamo, a Chinese online platform for data collection. We looked for potential participants at universities and invited them to participate in this online questionnaire. Each participant was told they would receive 2 CNY for completing the questionnaire. Participants indicated their agreement to participate through an electronic consent form at the beginning of the survey, then filled out the 5–10 min questionnaire and reported demographics. They were finally told to communicate with researchers in case of doubt. There are two criteria for screening participants. A survey was excluded if the response time was less than 180 s. In addition, we set up two attention-check items (participants were required to choose specified options based on the instructions). Participants who didn’t pass attention-check were excluded from data analyses. The study was done in according to the Declaration of Helsinki and approved by the university’s administrative units and the Research Ethics Committee.

### Measures

#### Personality trait of openness

We used the openness subscale of the Big Five Inventory [50] to assess openness. Sample items included “I see myself as someone who is inventive” and “I see myself as someone who has an active imagination”. Participants were asked to rate their agreement with each statement on a Likert scale ranging from 0 (strongly disagree) to 4 (strongly agree). The final score was the total of the item scores, with higher scores representing a higher level of openness. The Cronbach’s alpha in the current research was 0.82, and the KMO measure was 0.88 (*p* < 0.01).

#### Active and passive SNS use

Active and passive SNS use was assessed by the Active and Passive SNS Use Questionnaire [46], which consists of nine items and two dimensions: active SNS use (e.g., posting status updates) and passive SNS use (e.g., scrolling friends’ statuses, but not giving a “Like” or making a comment) (see Supplementary Materials for details). In the current research, participants were asked to rate the frequency of active or passive SNS use (1 = Never, 5 = Always). The final score was the mean of the item scores, with higher scores representing a higher frequency of active or passive SNS use. The questionnaire has good reliability and validity and is applicable to Chinese participants [51, 52]. In the research for developing this questionnaire [46], the original Cronbach’s alpha for the two dimensions was 0.83 and 0.84, respectively. In the current research, the Cronbach’s alpha coefficients for the two dimensions were 0.85 and 0.85, respectively. The KMO measure was 0.77 (*p* < 0.01). The results of the confirmatory factor analysis revealed that the questionnaire had good construct validity (χ^2^/df = 4.002, RMSEA = 0.076, CFI = 0.970, TLI = 0.955, SRMR = 0.056).

#### Self-evaluation

Self-evaluation was assessed by the Self-Evaluation Questionnaire [53], which consists of 15 items and three dimensions: warmth dimension (e.g., I think I am a sincere person), competence dimension (e.g., I think I am a smart person) and appearance dimension (e.g., I think I am a neat person) (see Supplementary Materials for details). In the current research, participants were asked to rate their agreement with each statement on a Likert scale ranging from 1 (strongly disagree) to 5 (strongly agree). The final score was the mean of the item scores, with higher scores representing a higher level of self-evaluation. The Cronbach’s alpha for the whole questionnaire was 0.88 and ranged between 0.67 and 0.80 for each dimension. The KMO measure was 0.88 (*p* < 0.01).

#### Social anxiety

Social anxiety was assessed by the social anxiety subscale of the Self-Consciousness Scale with six items [54] that were adapted from Fenigstein et al. [55]. Sample items included “Large groups make me nervous” and “It takes me time to get over my shyness in new situations”. Participants were asked to indicate the extent to which six items were like them, using the following response format: 0 = Not like me at all, 1 = A little like me, 2 = Somewhat like me, and 3 = A lot like me. The final score was the total of the item scores, with higher scores representing a higher level of social anxiety. The Cronbach’s alpha in the current research was 0.88, and the KMO measure was 0.88 (*p* < 0.01).

### Data analysis

SPSS25.0 and Mplus8.3 were used for data analysis. Data cleaning steps were performed preliminary to the analyses. Cronbach’s alpha, KMO test and confirmatory factor analysis were used to test the reliability and validity of the scales. The Harman single-factor test and confirmatory factor analysis were used to test whether the data had a serious common method bias. We computed the Skewness and Kurtosis values for the primary variables (i.e., openness, SNS use, self-evaluation, and social anxiety) to assess the normality of the distributions. The values were in the [− 1; 1] range, which indicated that the data distribution is normal. Therefore, there is no problem with using Pearson correlation and path analysis. We then made a bivariate association with all the primary variables. Zero means there is no correlation, where 1 means a complete or perfect correlation. A *p* < 0.05 was considered statistically significant for all tests. Finally, Mplus8.3 was used to build a multiple mediation model. To be specific, we used a path analysis to further explore the relationship between openness, active/passive SNS use, self-evaluation, and social anxiety. Multiple indicators were used to evaluate if the model was a good fit, including the Chi-square statistic, Root Mean Square Error of Approximation (RMSEA ≤ 0.08), Comparative Fit Index (CFI ≥ 0.95), Tucker-Lewis Index (TLI ≥ 0.95), Standardized Root Mean Squre Residual (SRMR ≤ 0.08). The mediating effect was examined with 5,000 bias-corrected bootstrapping and 95% percentile confidence intervals (CI). The effect is statistically significant if the CI does not include zero.

## Results

Table 1 displays the descriptive statistics and zero-order correlations for the major variables. As shown in Table 1, there is a gender difference in social anxiety. Therefore, gender was treated as a covariate in the subsequent analysis.

**Table 1:** Means, standard deviations and zero-order correlations among variables

Variable	Mean	*SD*	1	2	3	4	5	6	7
1 Gender	0.37	0.48	-						
2 Age	20.76	1.34	0.32**	-					
3 Openness	2.44	0.55	0.11*	–0.06	-				
4 Active SNS use	3.07	0.75	0.00	0.00	0.31**	-			
5 Passive SNS use	2.78	0.82	–0.02	0.05	–0.13**	–0.13**	-		
6 Self-evaluation	3.71	0.53	0.01	–0.03	0.44**	0.25**	–0.16**	-	
7 Social anxiety	2.44	0.77	–0.18**	–0.08	–0.29**	–0.16**	0.16**	–0.38**	-

* *p* < 0.05, ** *p* < 0.01; gender was coded “0” for females and “1” for males

### Discriminant validity analysis

We conducted a series of confirmatory factor analyses (CFAs) to determine the discriminant validity of measurements. Five variables were employed in the current study: openness, active SNS use, passive SNS use, self-evaluation and social anxiety. The five-factor measurement model was first tested as the baseline Model (M_0_). Then, according to the sizable significant correlations among variables (e.g., openness and self-evaluation), several other alternative models were further tested and compared with M_0_. As presented in Table 2, M_0_ exhibited an adequate fit to the data and provided significant improvement over the other alternative models. Given the results, we concluded that the constructs measured in our study have good distinctiveness.

**Table 2:** Comparison of measurement models

Model	χ^2^	*df*	Δχ^2^	RMSESA	CFI	TLI	SRMR
M_0_	1153.68	340		0.068	0.885	0.873	0.062
M_1_	1564.33	344	410.65**	0.082	0.828	0.811	0.075
M_2_	1671.18	344	517.50**	0.086	0.813	0.795	0.088
M_3_	2094.44	344	940.76**	0.099	0.754	0.729	0.096
M_4_	4779.22	350	3625.54**	0.156	0.377	0.327	0.141

** *p* < 0.01; M_0_: The hypothetical model; M_1_: Four factors (openness and self-evaluation combined into one factor); M_2_: Four factors (self-evaluation and social anxiety combined into one factor); M_3_: Four factors (two types of SNS use combined into one factor); M_4_: One factor (all variables combined into one factor)

### Common method bias test

Considering that all the data were obtained via participants’ self-reports, common method bias might have been present. In addition to using anonymous testing and reverse scoring, the Harman single-factor test and factor analysis were conducted in this study. Un-rotated exploratory factor analysis results extracted nine factors with eigenvalues greater than 1, and the first factor explained 22.62% of the total variation (less than 40%). Since the Harman single-factor test method may be insensitive, the method factor was added as a global factor based on the five-factor model. The five-factor model of the data fit well, but the model could not be fitted after adding the method factor. The above statistical tests showed that there was no obvious common method bias in this study.

### Mediation analysis

**Fig. 2:**
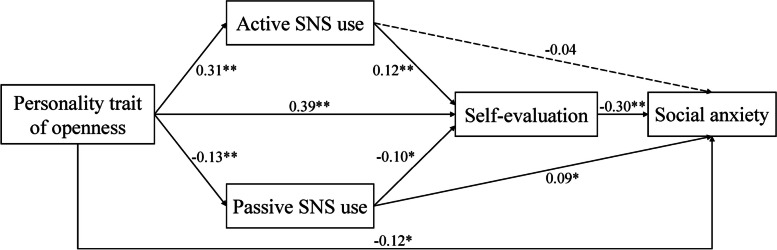
Standardized path coefficients for the structural equation model. * *p* < 0.05, ** *p* < 0.01

We tested the hypothetical model via path analysis using Mplus 8.3. The entire model showed an acceptable fit: χ^2^/*df* = 1.55, RMSEA = 0.033, CFI = 0.993, TLI = 0.974, SRMR = 0.022. Relationships among variables and the model’s standardized path coefficients are summarized in Fig. 2. Specifically, openness was positively related to active SNS use (*β* = 0.31, *p* < 0.01) and self-evaluation (*β* = 0.39, *p* < 0.01) but negatively related to passive SNS use (*β* = − 0.13, *p* < 0.01) and social anxiety (*β* = − 0.12, *p* < 0.05); active SNS use was positively related to self-evaluation (*β* = 0.12, *p* < 0.01); passive SNS use was positively related to social anxiety (*β* = 0.09, *p* < 0.05) but negatively related to self-evaluation (*β* = − 0.10, *p* < 0.05); and self-evaluation was negatively related to social anxiety (*β* = − 0.30, *p* < 0.01).

We then used bias-corrected bootstrapping to test the indirect effect (see Table 3). The results indicate that the mediation of openness–passive SNS use–social anxiety was significant (indirect effect = − 0.009, 95% CI [–0.027, − 0.001]). The chain mediation of openness–passive SNS use–self-evaluation–social anxiety was significant (indirect effect = − 0.003, 95% CI [–0.009, − 0.001]); the chain mediation of openness–active SNS use–self-evaluation–social anxiety was significant (indirect effect = − 0.009, 95% CI [–0.019, − 0.003]); and the mediation of openness–self-evaluation–social anxiety was significant (indirect effect = − 0.101, 95% CI [–0.145, − 0.067]). The total mediational effect size of openness on social anxiety was 56.84%, which was stronger than the direct effect of openness on social anxiety.

**Table 3:** Bootstrap analyses of the magnitude and statistical significance of mediating effects

	Effect	95% CI	
		Lower	Upper
Indirect effect (openness–passive SNS use–social anxiety)	–0.009	–0.027	–0.001
Indirect effect (openness–passive SNS use–self-evaluation–social anxiety)	–0.003	–0.009	–0.001
Indirect effect (openness–active SNS use–self-evaluation–social anxiety)	–0.009	–0.019	–0.003
Indirect effect (openness–self-evaluation–social anxiety)	–0.101	–0.145	–0.067
Direct effect	–0.101	–0.183	–0.014
Total effect	–0.234	–0.313	–0.147

In addition, as a robustness check, we switched the order of the two mediators (self-evaluation and active/passive SNS use), and the results did not support such a hypothetical model (see Supplementary Material for the results).

## Discussion

Based on the cognitive-behavioral model of social anxiety, the present study innovatively explored the relationship between openness and social anxiety from an online interaction perspective. In addition, it also clarified the specific relationships between openness and different forms of SNS use and the relationships between different forms of SNS use and self-evaluation. The results reveal that as the internet environment is gradually “socialized”, the connotation and extension of social activities have also changed dramatically, and personality differences that were previously considered distal factors of social anxiety are playing an increasingly important role.

The present study found that there was a negative correlation between openness and social anxiety, and Hypothesis 1 is confirmed. The results reaffirm the view that “specific personality traits accelerate the dysfunctional process” in the cognitive-behavioral model of social anxiety [43]. Individuals with low openness are less tolerant of unfamiliar situations and more focused on potential threat cues in social situations, which may make them less likely to benefit from daily interactions [56]. Thus, they seem more likely to produce dysfunctional beliefs. This reveals that although openness is more distantly related to the proximal factors of social anxiety disorder as traditionally viewed than personality traits such as neuroticism and extraversion, the cognitive orientation it represents may also be an important starting point for social anxiety. Recent cluster analytic studies of social anxiety have also shown that socially anxious individuals are not always introverted or highly neurotic [57]. Avoiding novel situations is a central characteristic of some social anxiety subgroups [57]. In addition, the present study also supports the notion that openness has an affect-amplifying function [26]. High openness may amplify the positive affective tendencies of individuals with a high degree of extroversion or agreeableness, resulting in a lower overall risk of social anxiety. This finding enlightens us that future research could adhere to the overall risk view and carefully investigate the synergistic and hindering effects among different personality traits to truly understand the status of personality in the mechanisms of social anxiety.

The present study found that self-evaluation mediates the relationship between openness and social anxiety, and Hypothesis 4 is confirmed. Previous studies have shown that openness is significantly and positively related to self-esteem, self-concept, and core self-evaluation [42, 58]. Our study further demonstrates that high openness helps individuals maintain rational and objective self-evaluation, which may make them not vulnerable to social anxiety in the Internet age of more social comparisons. In addition, previous research has shown that individuals with high openness have higher intelligence and cognitive ability, richer life experiences, and a slower cognitive decline in old age [59]. However, fewer researchers have linked this characteristic of openness to individuals’ self-evaluation. Considering the increasingly rich and novel means of social interaction, the advantages of highly open individuals in terms of intelligence and willingness to consider new things will become more prominent. This may imply that with the development of emerging information technologies (e.g., mobile internet), individuals with high openness will be more confident that they are capable of solving various problems and have a more positive self-concept; thus, openness may play an increasingly important role in the formation and development of social anxiety.

The present study found that active SNS use and self-evaluation play chain mediating roles in the relationship between openness and social anxiety; thus, Hypothesis 5 is confirmed. In contrast, active SNS use cannot independently mediate the relationship between openness and social anxiety, and Hypothesis 2 is not confirmed. Individuals with high openness actively use SNSs more frequently, which is similar to previous research findings (selection effect: the users’ predispositions affect SNS use) [60–62]. Our study further illustrates that although individuals with high openness are more open-minded about accepting and using the endless stream of new features on SNSs and actively use SNSs for self-expression and self-presentation, their ability to resist the risk of social anxiety will not be enhanced if such usage behaviors do not improve their self-evaluation. In other words, active SNS use may not directly alleviate social anxiety, and self-evaluation as a proximal factor of social anxiety plays an indispensable mediating role in the relationship between the two. This demonstrates that we should not only encourage individuals (especially high openness individuals) to actively use SNSs but also guide them to flexibly use the diverse and easy-to-use media features of SNSs to make a good impression. Only when their self-evaluation is effectively improved can they fully benefit from active SNS use.

The present study also found that passive SNS use can not only independently mediate the relationship between openness and social anxiety but also mediate the relationship between the two by the chain combination with self-evaluation; thus, Hypothesis 3 and Hypothesis 6 are confirmed. This indicates that high openness may reduce the frequency of individuals’ behavior in monitoring the updates of others, mitigating the harm caused to users by the flood of information through online interactions. As current SNSs promote the concept of visualization, their characteristics of information flooding remain unabated [63], which may lead to passive SNS users being more exposed to anxiety-provoking situations and, thus, becoming prone to social anxiety. Our study suggests that openness may be a key component in shaping people’s online social interaction experience, which can reduce the numbness and passivity caused by information flooding and prevent users from falling into the negative reinforcing spiral of “information browsing–negative self-evaluation–information browsing” [64], thus reduces the likelihood of social anxiety. This reveals that individuals with high openness may be more capable of correctly coping with the double-edged sword effect of SNSs, benefit from successful online interaction experiences, and form positive self-evaluations that are fed back into their real lives.

### Limitations and future research

Some limitations should be noticed. First, we adopted a cross-sectional research method; thus, the process model illustrated here is primarily based on theoretical inferences. In particular, based on social comparison theory and previous studies [46, 65], we regarded SNS usage behavior as an important environmental factor influencing self-evaluation. However, from the perspective of “evaluation-coping” [66], there is also the possibility that self-evaluation affects the use of SNSs. We conducted some additional statistical tests to try to rule out this possibility. Future studies should employ multifrequency and long-term longitudinal designs to further reveal the time-varying characteristics of the circular chain of SNS use, self-evaluation and social anxiety.

Second, the results of this study support the theoretical model tested, suggesting that openness is indeed negatively associated with social anxiety in the internet era. However, the proportion of SNS use with mediating effects was lower than expected, which may be due to some boundary conditions that were not taken into account. Although SNSs can provide users with media-rich social cues, not all high openness individuals value these cues [67]. For example, for high self-monitoring individuals, the attractiveness of using SNSs (both actively and passively) may be limited [67]. Future studies need to fully control such variables to obtain clearer conclusions.

Third, all data in this study were collected using the self-report method. To ensure that there was no obvious common method bias, we conducted a series of statistical tests. Future studies could combine self-assessment with other assessments (e.g., objective data from internet devices) to obtain more objective data.

### Practical implications

The study has some significant practical implications. Taking openness into account may be useful for the clinical treatment of social anxiety. The therapist can get to know the patient from this starting point and set appropriate treatment strategies and targets. For example, in response to the characteristics of cognitive closure in individuals with low openness, therapists can enhance their tolerance to unfamiliar situations through cognitive behavioral therapy, thereby improving the quality of interpersonal interactions. Moreover, it is essential to properly understand the two sides of SNS use. This study considered the relationship between SNS use and mental health from two dimensions: active use and passive use. Specifically, active SNS use may be beneficial in alleviating social anxiety, as opposed to passive SNS use. This contributes to a more comprehensive and deeper understanding of SNS use. The community should guide people to use SNSs more properly, not less.

## Conclusion

This study examined the relationship between openness and social anxiety in the Internet era based on the cognitive-behavioral model of social anxiety. Findings show that openness is negatively associated with social anxiety, and active/passive SNS use and self-evaluation play chain mediating roles in this relationship. These results provide insights into the clinical treatment of social anxiety and how to benefit from online interactions.

### Supplementary Information

**Additional file 1.**

## Data Availability

The datasets used and/or analysed during the current study available from the corresponding author on reasonable request.

## References

[1] Stein MB, Stein DJ (2008). Social anxiety disorder. Lancet.

[2] Zinbarg RE, Uliaszek AA, Adler JM (2008). The role of personality in psychotherapy for anxiety and depression. J Pers.

[3] Newby J, Pitura VA, Penney AM, Klein RG, Flett GL, Hewitt PL (2017). Neuroticism and perfectionism as predictors of social anxiety. Pers Indiv Differ.

[4] Becker MW, Alzahabi R, Hopwood CJ (2013). Media multitasking is associated with symptoms of depression and social anxiety. Cyberpsych Beh Soc N.

[5] Aurora P, Coifman KG (2021). Unpacking social avoidance and substance use in social anxiety: does extraversion explain behavior variability?. J Psychopathol Behav Assess.

[6] Schlenker BR, Leary MR (1982). Social anxiety and self-presentation: a conceptualization and model. Psychol Bull.

[7] Andersen AM, Bienvenu OJ (2011). Personality and psychopathology. Int Rev Psychiatr.

[8] Digman JM (1997). Higher-order factors of the big five. J Pers Soc Psychol.

[9] García LF, Aluja A, García Ó, Cuevas L (2005). Is openness to experience an Independent personality dimension? Convergent and discriminant validity of the openness domain and its NEO-PI-R facets. J Indiv Differ.

[10] Watson D, Naragon-Gainey K (2014). Personality, emotions, and the emotional disorders. Clin Psychol Sci.

[11] Cohrs JC, Kämpfe-Hargrave N, Riemann R (2012). Individual differences in ideological attitudes and prejudice: evidence from peer-report data. J Pers Soc Psychol.

[12] Saroglou V (2002). Religion and the five factors of personality: a meta-analytic review. Pers Indiv Differ.

[13] Jost JT, Glaser J, Kruglanski AW, Sulloway FJ (2003). Political Conservatism as motivated social cognition. Psychol Bull.

[14] Łakuta P (2019). Personality trait interactions in risk for and protection against social anxiety symptoms. J Pers.

[15] DeYoung CG, Cooper ML, Larsen RJ (2015). Openness/intellect: a dimension of personality reflecting cognitive exploration. APA handbook of personality and social psychology: personality processes and individual differences(Vol. 4).

[16] Lü W. Social stress reaction and autonomic nervous regulation mechanism of individuals with positive personality traits. PhD thesis. Shaanxi Normal University, School of Psychology; 2014.

[17] Gerra G, Zaimovic A, Zambelli U, Timpano M, Reali N, Bernasconi S (2000). Neuroendocrine responses to psychological stress in adolescents with anxiety disorder. Neuropsychobiology.

[18] Utz S (2015). The function of self-disclosure on social network sites: not only intimate, but also positive and entertaining self-disclosures increase the feeling of connection. Comput Hum Behav.

[19] Liu D, Campbell WK (2017). The big five personality traits, big two metatraits and social media: a meta-analysis. J Res Pers.

[20] Ross C, Orr ES, Sisic M, Arseneault JM, Simmering MG, Orr RR (2009). Personality and motivations associated with Facebook use. Comput Hum Behav.

[21] Amichai-Hamburger Y, Vinitzky G (2010). Social network use and personality. Comput Hum Behav.

[22] Moore K, McElroy JC (2012). The influence of personality on Facebook usage, wall postings, and regret. Comput Hum Behav.

[23] Wilson K, Fornasier S, White KM (2010). Psychological predictors of young adults’ use of social networking sites. Cyberpsych Beh Soc N.

[24] Hughes DJ, Rowe M, Batey M, Lee A (2012). A tale of two sites: Twitter vs. Facebook and the personality predictors of social media usage. Comput Hum Behav.

[25] Verduyn P, Lee DS, Park J, Shablack H, Orvell A, Bayer J (2015). Passive Facebook usage undermines affective well-being: experimental and longitudinal evidence. J Exp Psychol Gen.

[26] McCrae RR, Costa PT (1991). Adding liebe und arbeit: the full five-factor model and well-being. Pers Soc Psychol Bull.

[27] Lee E, Ahn J, Kim YJ (2014). Personality traits and self-presentation at Facebook. Pers Indiv Differ.

[28] Seidman G (2013). Self-presentation and belonging on Facebook: how personality influences social media use and motivations. Pers Indiv Differ.

[29] Carpenter JM, Green MC, LaFlam J (2011). People or profiles: individual differences in online social networking use. Pers Indiv Differ.

[30] Burke M, Marlow C, Lento T. Social network activity and social well-being. In: Proceedings of the SIGCHI Conference on Human Factors in Computing Systems, CHI: 2010; 1909–1912.

[31] Thorisdottir IE, Sigurvinsdottir R, Asgeirsdottir BB, Allegrante JP, Sigfusdottir ID (2019). Active and passive social media use and symptoms of anxiety and depressed mood among Icelandic adolescents. Cyberpsych Beh Soc N.

[32] Verduyn P, Ybarra O, Résibois M, Jonides J, Kross E (2017). Do social network sites enhance or undermine subjective well-being? A critical review. Soc Iss Policy Rev.

[33] O’Day EB, Heimberg RG (2021). Social media use, social anxiety, and loneliness: a systematic review. Comput Hum Behav Rep.

[34] Lee BW, Stapinski LA (2012). Seeking safety on the internet: relationship between social anxiety and problematic internet use. J Anxiety Disord.

[35] Shane-Simpson C, Manago A, Gaggi N, Gillespie-Lynch K (2018). Why do college students prefer Facebook, Twitter, or Instagram? Site affordances, tensions between privacy and self-expression, and implications for social capital. Comput Hum Behav.

[36] Collins RL (1996). For better or worse: the impact of upward social comparison on self-evaluations. Psychol Bull.

[37] Tandoc EC, Ferrucci P, Duffy M (2015). Facebook use, envy, and depression among college students: is facebooking depressing?. Comput Hum Behav.

[38] Shaw AM, Timpano KR, Tran TB, Joormann J (2015). Correlates of Facebook usage patterns: the relationship between passive Facebook use, social anxiety symptoms, and brooding. Comput Hum Behav.

[39] Rapee RM, Heimberg RG (1997). A cognitive-behavioral model of anxiety in social phobia. Behav Res Ther.

[40] Harter S (2015). The construction of the self: Developmental and sociocultural foundations.

[41] Li HY, Cui YW, He YS, Xiao P, Wang L (2014). A review on the research of the core self-evaluation. Stud Psychol Behav.

[42] Judge TA, Van Vianen AEM, De Pater IE (2004). Emotional stability, core self-evaluations, and job outcomes: a review of the evidence and an agenda for future research. Hum Perform.

[43] Clark DM, Wells A, Heimberg G, Liebowitz MR, Hope D, Scheier F (1995). A cognitive model of social phobia. Social phobia: diagnosis, assessment, and treatment.

[44] Moscovitch DA (2009). What is the core fear in social phobia? A new model to facilitate individualized case conceptualization and treatment. Cogn Behav Pract.

[45] Vogel EA, Rose JP, Roberts LR, Eckles K (2014). Social comparison, social media, and self-esteem. Psychol Pop Media.

[46] Ding Q. The impact of social network sites use on adolescents’ self-evaluation: Base on social comparison theory. PhD thesis. Central China Normal University, School of Psychology; 2017.

[47] Liu QQ, Niu GF, Fan CY, Zhou ZK (2017). Passive use of social network site and its relationships with self-esteem and self-concept clarity: a moderated mediation analysis. Acta Psychol Sin.

[48] Yang CC. It makes me feel good: A longitudinal, mixed-methods study on college freshmen’s Facebook self-presentation and self-development. PhD thesis. University of Wisconsin-Madison; 2014.

[49] Lian SL, Sun XJ, Yang XJ, Zhou ZK (2020). The effect of adolescents’ active social networking site use on life satisfaction: the sequential mediating roles of positive feedback and relational certainty. Curr Psychol.

[50] John OP, Srivastava S, Pervin L, John O (1999). The big five trait taxonomy: history, measurement, and theoretical perspectives. Handbook of personality: theory and research.

[51] Hou J, Tian S, Sun XY, Xie SY, Cao QX, Wang XY (2020). The influence of big five personality on the use of social networking sites: the mediating role of narcissism. Chin J Clin Psychol.

[52] Ou XY. The relationship between the use of social networking sites and mental health of adolescents: The role of upward social comparison and friendship quality. master’s thesis. Guangxi Mingzu University, School of Education Science; 2020.

[53] Wang J. Effect of self-impression evaluation on emotional expressions of senior high school students and its intervention. master’s thesis. Central China Normal University, School of Psychology; 2019.

[54] Scheier MF, Carver CS (1985). The self-consciousness scale: a revised version for use with general populations. J Appl Soc Psychol.

[55] Fenigstein A, Scheier MF, Buss AH (1975). Public and private self-consciousness: Assessment and theory. J Consult Clin Psych.

[56] McCrae RR (1994). Openness to experience: expanding the boundaries of factor V. Eur J Pers.

[57] Costache ME, Frick A, Månsson K, Engman J, Faria V, Hjorth O (2020). Higher- and lower-order personality traits and cluster subtypes in social anxiety disorder. PLoS ONE.

[58] Robins RW, Tracy JL, Trzesniewski K, Potter J, Gosling SD (2001). Personality correlates of self-esteem. J Res Pers.

[59] Graham EK, Lachman ME (2012). Personality stability is associated with better cognitive performance in adulthood: are the stable more able?. J Gerontol B.

[60] Correa T, Hinsley AW, de Zuniga HG (2010). Who interacts on the web? The intersection of users’ personality and social media use. Comput Hum Behav.

[61] Guadagno RE, Okdie BM, Eno CA (2008). Who blogs? Personality predictors of blogging. Comput Hum Behav.

[62] Trepte S, Reinecke L (2013). The reciprocal effects of social network site use and the disposition for self-disclosure: a longitudinal study. Comput Hum Behav.

[63] Guo Y, Lu Z, Kuang H, Wang C (2020). Information avoidance behavior on social network sites: information irrelevance, overload, and the moderating role of time pressure. Int J Inform Manage.

[64] Slater MD (2007). Reinforcing spirals: the mutual influence of media selectivity and media effects and their impact on individual behavior and social identity. Commun Theor.

[65] Solomon M. Social media and self-evaluation: The examination of social media use on identity, social comparison, and self-esteem in young female adults. PhD thesis. William James College; 2016.

[66] Kammeyer-Mueller JD, Judge TA, Scott BA (2009). The role of core self-evaluations in the coping process. J Appl Psychol.

[67] Lee DKL, Borah P (2020). Self-presentation on Instagram and friendship development among young adults: a moderated mediation model of media richness, perceived functionality, and openness. Comput Hum Behav.

