# Supplementary material for: The relationship between openness and social anxiety: the chain mediating roles of social networking site use and self-evaluation
Source: BMC Psychol. 2023 Nov 13;11:391. doi: 10.1186/s40359-023-01412-y (PMC10644632; doi:10.1186/s40359-023-01412-y)
Supplement: Supplementary file 1 — Additional file 1. [file 40359_2023_1412_MOESM1_ESM.docx]

**Supplementary Materials for**

The Relationship between Openness and Social Anxiety: The Chain Mediating Roles of Social Networking Site Use and Self-evaluation

**This file includes:**

Questionnaire details

Supplementary mediation analysis (as a robustness check)

**Questionnaire details:**

**Active and Passive SNS Use Questionnaire** (originally in Chinese, translated by the author).

1. Posting status updates.

2. Posting photos.

3. Commenting on friends’ statuses.

4. Commenting on friends’ photos.

5. Giving friends’ statuses or photos a “Like”.

6. Scrolling friends’ statuses, but not giving a “Like” or making a comment.

7. Viewing friends’ photos, but not giving a “Like” or making a comment.

8. Clicking on the links shared by friends, but not retweeting, giving a “Like” or making a comment.

9. Browsing friends’ homepages, but not interacting with them.

**Self-Evaluation Questionnaire** (originally in Chinese, translated by the author).

1. I think I am a friendly person.

2. I think I am a warm person.

3. I think I am a capable person.

4. I think I am a sincere person.

5. I think I am an incompetent person.

6. I think I am a cold person.

7. I think I am a lazy person.

8. I think I am a selfish person.

9. I think I am a smart person.

10. I think I am a hypocritical person.

11. I think I am a clumsy person.

12. I think I am a good-looking person.

13. I think I am a neat person.

14. I think I am a healthy person.

15. I think I am a dowdy person.

**Supplementary mediation analysis (as a robustness check):**

| **Table 1** Indirect effect tests | | | |
| --- | --- | --- | --- |
|  | effect | 95% CI | |
|  |  | Lower | Upper |
| Hypothesis 5  openness–active SNS use–self-evaluation–social anxiety | –0.009 | –0.019 | –0.003 |
| *Reverse causality*  openness–self-evaluation–active SNS use–social anxiety | –0.002 | –0.009 | 0.002 |
| Hypothesis 6  openness–passive SNS use–self-evaluation–social anxiety | –0.003 | –0.009 | –0.001 |
| *Reverse causality*  openness–self-evaluation–passive SNS use–social anxiety | –0.004 | –0.012 | –0.001 |
